# Supplementary material for: The effects of genital myiasis on the diversity of the vaginal microbiota in female Bactrian camels
Source: BMC Vet Res. 2022 Mar 5;18:87. doi: 10.1186/s12917-022-03189-5 (PMC8897907; doi:10.1186/s12917-022-03189-5)
Supplement: Supplementary file 5 — Additional file 5. [file 12917_2022_3189_MOESM5_ESM.zip › MPL201709200_16s_yy/Treat1/css/js_css/jsPDF-master.zip!/jsPDF-master/docs/PubSub.html]

PubSub - Documentation


## Home

### Classes

- FontObject
- jsPDF
- PubSub

### Global

- addFont
- addMetadata
- addPage
- CapJoinStyles
- circle
- CssColors
- ellipse
- getFontList
- html2pdf
- http
- lines
- lstext
- output
- rect
- requirejs
- reset
- roundedRect
- save
- setDisplayMode
- setDrawColor
- setFillColor
- setFont
- setFontSize
- setFontStyle
- setLineCap
- setLineJoin
- setLineWidth
- setPage
- setProperties
- setTextColor
- text
- toUrl
- triangle
- triggerEvent

# PubSub

## PubSub

#### new PubSub()

Source:
:   - jspdf.js, line 105

jsPDF's Internal PubSub Implementation.
See mrrio.github.io/jsPDF/doc/symbols/PubSub.html
Backward compatible rewritten on 2014 by
Diego Casorran, https://github.com/diegocr

  

Documentation generated by JSDoc 3.4.2 on Sat Oct 08 2016 21:42:26 GMT+0100 (BST) using the docdash theme.
